# Increased production of periplasmic proteins in *Escherichia coli* by directed evolution of the translation initiation region

**DOI:** 10.1186/s12934-020-01339-8

**Published:** 2020-04-07

**Authors:** Kiavash Mirzadeh, Patrick J. Shilling, Rageia Elfageih, Alister J. Cumming, Huanhuan L. Cui, Maja Rennig, Morten H. H. Nørholm, Daniel O. Daley

**Affiliations:** 1 Department of Biochemistry and Biophysics, Stockholm University, Stockholm, Sweden; 2 CloneOpt AB, Stockholm, Sweden; 3 Department of Medicine (Solna), Division of Microbial Pathogenesis, BioClinicum, Karolinska Institutet, Stockholm, Sweden; 4 Novo Nordisk Foundation Center for Biosustainability, Technical University of Denmark, Kgs. Lyngby, Denmark

**Keywords:** Protein secretion, Signal peptide, Recombinant protein expression, Bacteria, Translation initiation, Directed evolution

## Abstract

**Background:**

Recombinant proteins are often engineered with an N-terminal signal peptide, which facilitates their secretion to the oxidising environment of the periplasm (gram-negative bacteria) or the culture supernatant (gram-positive bacteria). A commonly encountered problem is that the signal peptide influences the synthesis and secretion of the recombinant protein in an unpredictable manner. A molecular understanding of this phenomenon is highly sought after, as it could lead to improved methods for producing recombinant proteins in bacterial cell factories.

**Results:**

Herein we demonstrate that signal peptides contribute to an unpredictable translation initiation region. A directed evolution approach that selects a new translation initiation region, whilst leaving the amino acid sequence of the signal peptide unchanged, can increase production levels of secreted recombinant proteins. The approach can increase production of single chain antibody fragments, hormones and other recombinant proteins in the periplasm of *E. coli.*

**Conclusions:**

The study demonstrates that signal peptide performance is coupled to the efficiency of the translation initiation region.

## Background

Bacterial cell factories are widely used in the biotech and pharmaceutical industries for the production of high-value recombinant proteins. Classic examples include industrial enzymes, hormones and antibody fragments, which generate billions of dollars in revenue annually [1, 2]. These recombinant proteins are typically engineered with an N-terminal signal peptide so that they will be secreted out of the bacterial cytoplasm [3, 4]. For industrial enzymes, which are usually produced in gram-positive bacteria such as *Bacillus subtilis*, secretion from the cytoplasm to the culture supernatant simplifies purification and downstream processing. For hormones and antibody fragments, which are usually produced in gram-negative bacteria like *Escherichia coli*, secretion from the cytoplasm to the oxidising environment of the periplasm is necessary for the formation of disulfide bonds that are essential for protein folding and activity [5, 6].

Secretion out of the bacterial cytoplasm is usually mediated by the general secretion pore (Sec) [7, 8]. Sec is a major hub for protein trafficking as it inserts proteins into the cytoplasmic membrane, and secretes proteins to the envelope and beyond. Secreted proteins are typically targeted to Sec by a cleavable N-terminal signal peptide. Signal peptides vary in length and amino acid sequence, but have a distinctive tripartite structure that includes a positively-charged N-terminal region, a hydrophobic core, and a polar C-terminal cleavage site that contains the signal peptidase recognition site (Ala-X-Ala) [9, 10]. They also have a distinctive codon usage, which includes a biased use of the AAA (Lys) codon at the second position, and a high frequency of non-optimal codons [11–15]. It has been suggested that the signal peptide slows folding of the protein in the cytoplasm and targets it to Sec in a predominantly unfolded confirmation [16]. Upon arrival at Sec the signal peptide also promotes binding to the SecA chaperone, thereby allosterically activating Sec for protein secretion [17]. Given these multiple roles it is likely that signal peptides have co-evolved with the protein that they translocate, as well as with the secretion machinery.

Signal peptides have unpredictable effects on the production yields of recombinant proteins. For example, a signal peptide that supports a high-level of protein synthesis and secretion for one recombinant protein often supports a low-level of protein synthesis and secretion for another [3, 4, 18]. Herein we refer to this phenomenon as signal peptide performance. Since it is not possible to predict how well a signal peptide will perform with a given recombinant protein, it is common practice to screen signal peptide-libraries for one that supports a high-level of protein synthesis and secretion [3, 4, 18]. This approach is both time-consuming and expensive. A molecular understanding of signal peptide performance is therefore highly sought after, as it could lead to new methods for (1) identifying suitable signal peptides, and (2) rationally engineering signal peptides that increase production yields in bacterial cell factories.

**Fig. 1:**
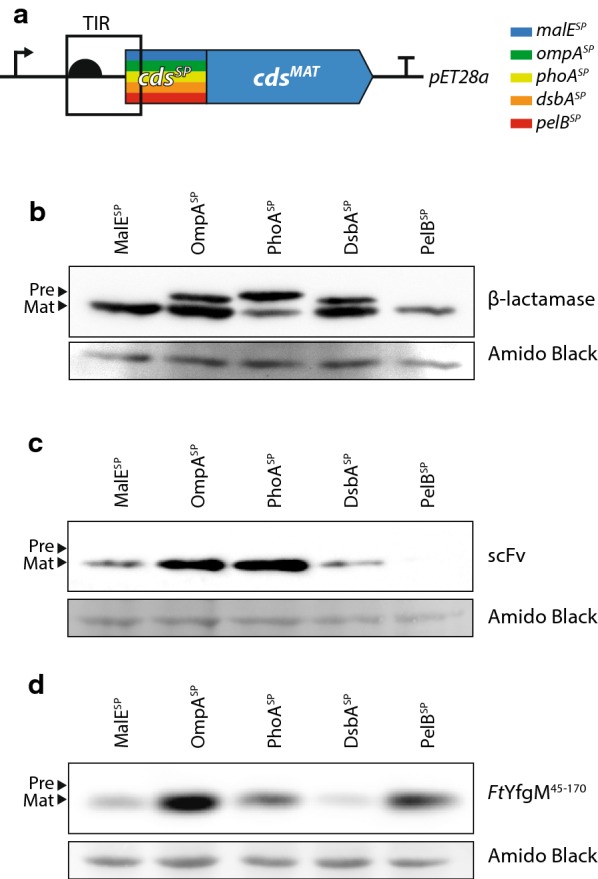
A comparison of commonly used signal peptides. **a** An overview of the expression cassettes used in this experiment. The TIR is represented by the boxed area, which extends from the Shine-Dalgarno to the fifth codon of the signal sequence. The coding sequences (CDS) for five commonly used signal peptides (MalE^SP^, OmpA^SP^, PhoA^SP^, DsbA^SP^, PelB^SP^) were cloned into the pET28a vector, upstream of the mature CDS for β-lactamase, scFv^HER2^ or *Ft*YfgM^45−170^. Protein production was induced for 2 h, then a volume of cells corresponding to 0.2 OD_600_ units of cells were harvested, separated by a 12% SDS-PAGE and protein levels determined by immuno-blotting with antisera to β-lactamase **b**, or the poly-Histidine tag of scFv^HER2^**c** and *Ft*YfgM^45–170^**d**. To ensure that protein loading was consistent between the samples, the membranes were stained with Amido black after immuno-detection. ‘Pre’ denotes the precursor form of the protein, which contains the signal sequence and is presumed to be in the cytoplasm. ‘Mat’ precursor denotes the mature form, which is presumed to be in the periplasm as the signal peptide has been cleaved

In this study we have noted that signal peptides have a significant impact on translation initiation; the rate-limiting step in protein synthesis [19–23]. The efficiency of translation initiation is dependent on the nucleotide sequence of the translation initiation region (TIR) [24–27], a stretch of approximately thirty nucleotides that extends from the Shine-Dalgarno region to the fifth codon of the coding sequence (i.e. the first ribosomal footprint) [28]. When signal peptides are fused to recombinant proteins, the TIR is formed ad hoc by genetically fusing the 5′ UTR encoded in the plasmid to the coding sequence of the signal peptide (Fig. 1a). Thus each time a different signal peptide is fused, a different TIR is generated. Herein we demonstrate that TIRs formed by genetic fusion are not optimal for protein production, and that protein production yields can be increased using a directed evolution approach that selects a new translation initiation region, whilst leaving the amino acid sequence of the signal peptide unchanged. Taken together the data indicate that signal sequence performance is tightly coupled to the nucleotide sequence of the TIR.

## Results

### Production of periplasmic proteins with commonly used signal peptides

Five signal peptides that are commonly used for the production of recombinant proteins in the periplasm of *E. coli* were selected (MalE^SP^, OmpA^SP^, PhoA^SP^, DsbA^SP^ and PelB^SP^; Table 1). The coding sequences for these signal peptides were cloned into the commonly used pET28a expression plasmid, upstream of the coding sequence for β-lactamase (Fig. 1a). To determine how efficiently the signal peptides supported the synthesis and secretion of β-lactamase the expression plasmids were transformed into the *E. coli* strain BL21(*DE3*) *pLysS* and a mild induction protocol was used to initiate transcription (0.05 mM IPTG for 2 h at 30 °C). Following the induction period, whole cells were collected, and proteins were separated by SDS-PAGE and immuno-blotted, so that the secreted (Mature) and non-secreted (Precursor) β-lactamase could be distinguished. The experiment indicated that MalE^SP^, OmpA^SP^, PhoA^SP^, DsbA^SP^ supported a comparatively high-level of β-lactamase synthesis, whereas PelB^SP^ did not (Fig. 1b). Moreover, MalE^SP^ and PelB^SP^ were effective in supporting the secretion of β-lactamase to the periplasm, as only the Mature sized protein was detected. In contrast OmpA^SP^, PhoA^SP^ and DsbA^SP^ were less effective in supporting the secretion of β-lactamase as there was a prominent Precursor band, indicating that the signal peptide was not cleaved. The latter observation most likely means that the β-lactamase was not secreted to the periplasm.

**Table 1:** Signal peptides used in this study

Signal peptide	Sequence	Origin
MalE^SP^	MKIKTGARILALSALTTMMFSASALA	*Escherichia coli*
OmpA^SP^	MKKTAIAIAVALAGFATVAQA	*Escherichia coli*
PhoA^SP^	MKQSTIALALLPLLFTPVTKA	*Escherichia coli*
DsbA^SP^	MKKIWLALAGLVLAFSASA	*Escherichia coli*
PelB^SP^	MKYLLPTAAAGLLLLAAQPAMA	*Erwinia carotovora*

To evaluate the performance of the signal peptides with other recombinant proteins, we fused them to a single chain variable fragment that recognises the human epidermal growth factor receptor protein 2 protein (scFv^HER2^) and a soluble fragment of the periplasmic chaperone YfgM from *Francisella tularensis* (*Ft*YfgM^45−170^). Again there were differences in synthesis levels of scFv^HER2^ and *Ft*YfgM^45−170^ when using different signal peptides (Fig. 1c, d). Taken together, these observations demonstrate that signal peptide performance is varied and unpredictable during the synthesis and secretion of recombinant periplasmic proteins. This conclusion is supported by a large body of published work, however a molecular explanation for the phenomenon remains elusive (reviewed in [3, 4]).

### Signal peptide performance is coupled to translation initiation

**Fig. 2:**
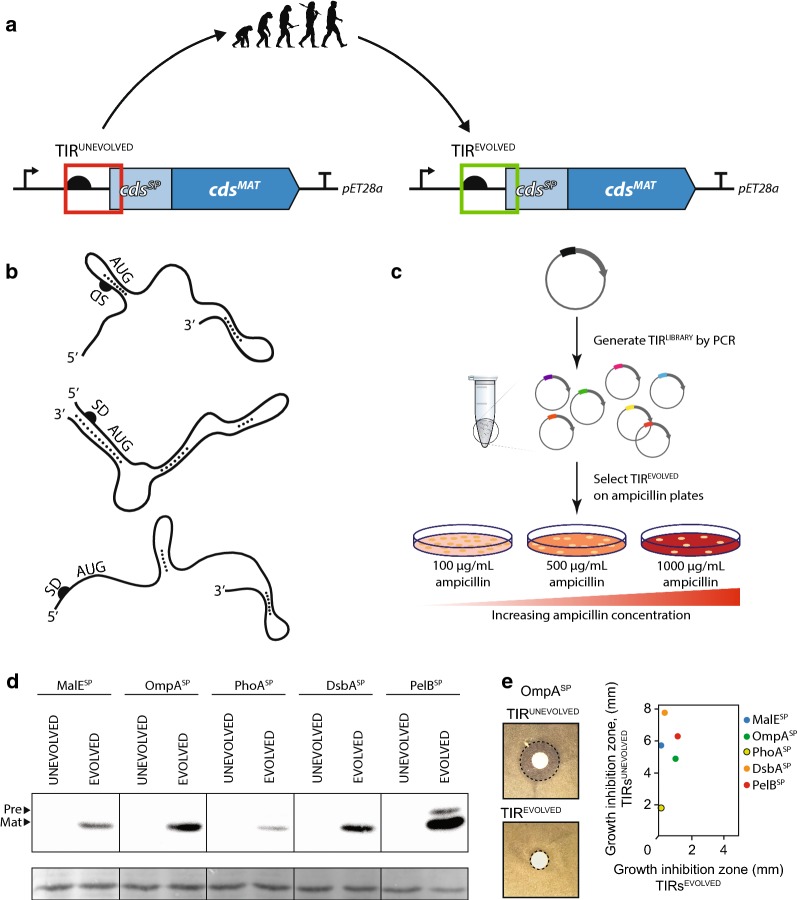
Improved signal peptide performance following directed evolution of the TIR **a** A directed evolution approach was used to convert a TIR^UNEVOLVED^ to a TIR^EVOLVED^. The TIR is defined as the region from the Shine-Dalgarno (half-dome) to codon 5 of the signal peptide. **b** mRNA has a high propensity to form structures, thus a TIR^UNEVOLVED^ can be sequestered into short- (top) or long-range structures (middle). Directed evolution should select a TIR^EVOLVED^ that is less structured and more accessible to the ribosome during translation initiation (bottom). **c** An overview of the directed evolution process. A TIR^LIBRARY^ was constructed by completely randomising the six nucleotides immediately upstream of the AUG start codon, and partially randomising the six nucleotides immediately downstream of the AUG start codon (allowing synonymous codons changes only). The TIR^LIBRARY^ was transformed into *E. coli* BL21(*DE3*) pLysS and plated on increasing concentrations of ampicillin. A TIR^EVOLVED^ was identified on the plate containing the highest concentration of ampicillin relative to the TIR^UNEVOLVED^ variant. **d** β-lactamase production levels from TIR^UNEVOLVED^/TIR^EVOLVED^ pairs was assessed by immuno-blotting. In this experiment, β-lactamase production was induced for 2 h, then a volume of cells corresponding to 0.2 OD_600_ units of cells were harvested, separated by a 12% SDS-PAGE and protein levels were determined by immuno-blotting with antisera to β-lactamase. To ensure that protein loading was consistent between the samples, the membrane was stained with Amido black after immuno-detection. ‘Pre’ denotes the precursor form of the protein, which contains the signal peptide fused version of β-lactamase, which we presume to be in the cytoplasm as the signal peptide is still present. ‘Mat’ precursor denotes the mature version of β-lactamase, which presumably is in the periplasm as the signal peptide has been cleaved. (E) β-lactamase activity from TIR^UNEVOLVED^/TIR^EVOLVED^ pairs was assessed using the disc diffusion assay. Here a filter disc containing 2 mg of ampicillin was placed on top of an LB-agar plate containing a lawn of bacteria expressing β-lactamase from either a TIR^UNEVOLVED^ or a TIR^EVOLVED^. The diameter of the growth-inhibition zone was measured for each experiment. In all cases, a TIR^EVOLVED^ conferred more resistant to ampicillin than TIR^UNEVOLVED^

In our recombinant expression plasmids, the TIR was formed by fusing the 5′UTR from the expression plasmid with the coding sequence of the signal peptide (Fig. 2a, b). Thus each time a different signal peptide was used a different TIR was generated. We refer to each of these TIRs as unevolved (i.e. TIR^UNEVOLVED^) as they were formed by ad hoc genetic fusion rather than co-evolution with the host cell ribosomes. We hypothesised that a TIR^UNEVOLVED^ would not be optimal for protein production.

We employed a directed evolution approach to select a TIR that was more suitable for protein production. In the directed evolution experiment, TIR^LIBRARIES^ were created from the expression plasmids encoding the MalE^SP^, OmpA^SP^, PhoA^SP^, DsbA^SP^ and PelB^SP^ fused to β-lactamase. In the design of the TIR^LIBRARIES^, the six nucleotides immediately upstream from the AUG start codon were completely randomised, and the six nucleotides immediately downstream were randomised with synonymous codon changes only (Fig. 2c) [29, 30]. Each TIR^LIBRARY^ theoretically contained > 18,000 expression plasmids with a different TIR. The TIR^LIBRARIES^ were transformed into BL21(*DE3*) *pLysS* and plated onto LB agar containing 0.05 mM IPTG and different concentrations of ampicillin (Fig. 2c). Twenty colonies that were resistant to a high concentration of ampicillin were initially selected and sequenced (Additional file 1: Fig. 1). The expression of β-lactamase from each unique TIR was compared by immuno-blotting (Additional file 1: Fig. 2). Those expression plasmids that supported a high level of β-lactamase were selected. The evolved TIRs (TIR^EVOLVED^) are presented in Table 2. Each TIR^EVOLVED^ was then re-constructed in a fresh expression vector, and the production levels of β-lactamase again compared by immuno-blotting. After a 2-h induction period we observed that more β-lactamase was synthesised when a TIR^EVOLVED^ was used (Fig. 2d). Note that production of β-lactamase from each TIR^UNEVOLVED^ was undetectable on these blots because the difference from the TIR^EVOLVED^ was too large to capture at this time point (see below). Consistent with this observation, disc diffusion assays confirmed that the TIR^EVOLVED^ supported a higher level of resistance to ampicillin than the TIR^UNEVOLVED^ (Fig. 2e). We could not explain the difference in production levels from the TIR^UNEVOLVED^/TIR^EVOLVED^ pairs using mRNA structure predictions. Nevertheless, the data underscores the fact that the synthesis of secreted recombinant proteins in bacterial cell factories is tightly coupled to the nucleotide sequence of the TIR.

**Table 2:**
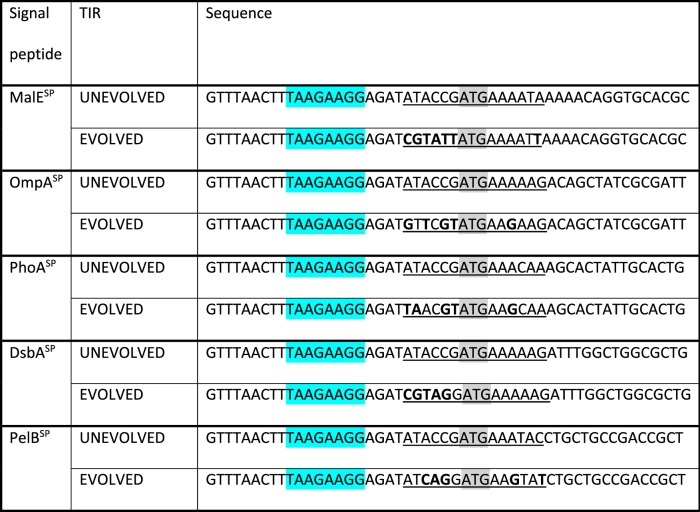
Nucleotide sequences of the TIR^UNEVOLVED^ and corresponding TIR^EVOLVED^ used in this study. The TIR is defined as the region from the Shine-Dalgarno to codon five of the signal peptide

^1^Underlined region was randomised during the directed evolution process

^2^Nucleotides marked in bold text were changed in TIR^SYN_EVOLVED^

^3^Shine-Dalgarno sequence marked in blue ^4^Start methionine (ATG) marked in grey

### Secretion is not a bottle-neck when synthesis levels are increased

**Fig. 3:**
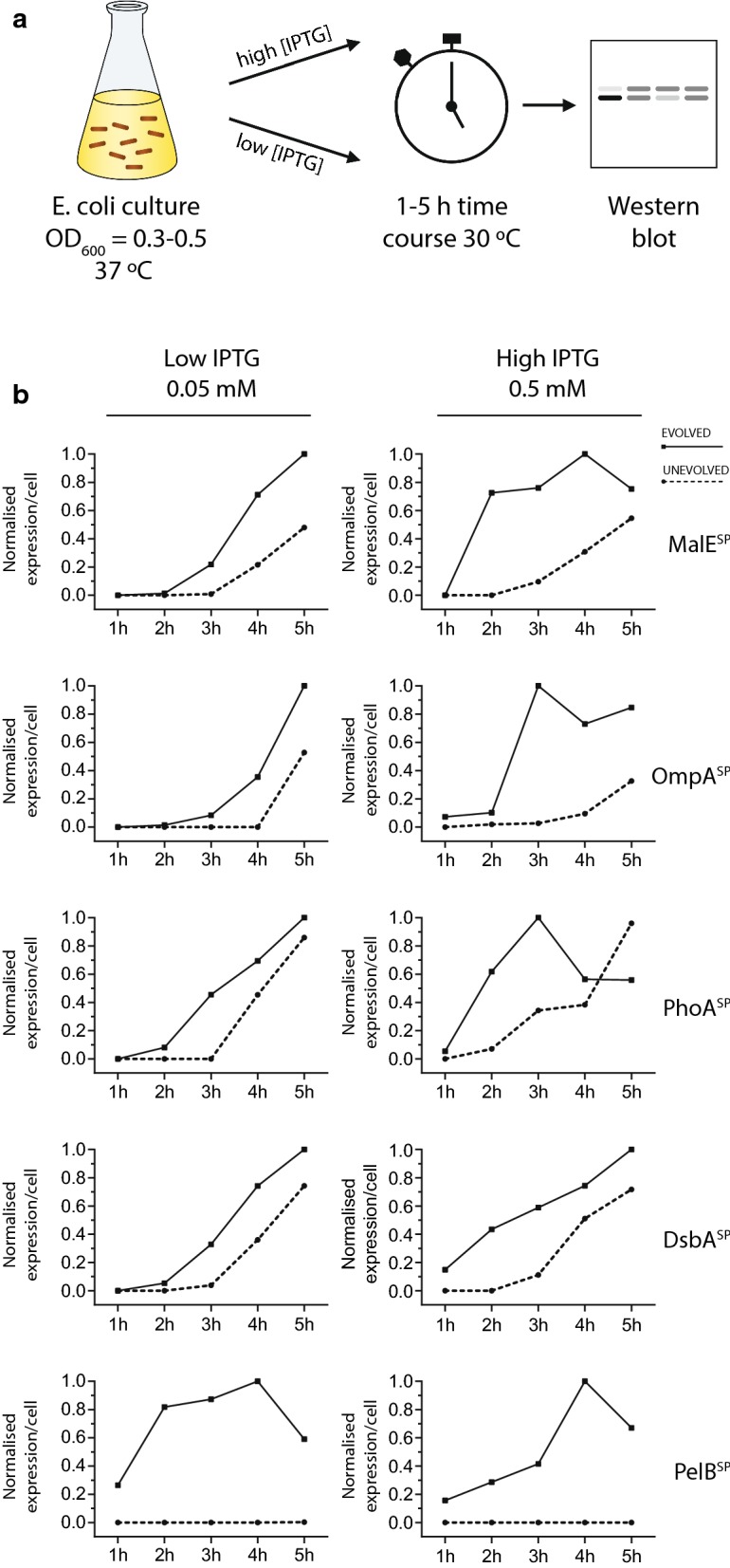
Time-course analysis of β-lactamase production. **a** An illustration of the experimental workflow used. **b** At each time point, a volume of cells was extracted, then separated by SDS-PAGE and immuno-blotted with antisera to β-lactamase. Band intensities were obtained from immuno-blots by densitometric analysis and normalised to the highest-value

In the previous series of experiments a mild induction protocol was used (0.05 mM IPTG for 2 h at 30 °C), so that differences in protein synthesis could be assessed in the absence of a metabolic load on the cell. The concern about metabolic load largely related to the Sec translocon, which is believed to be a bottleneck in the production of periplasmic proteins [31, 32]. When production levels of periplasmic proteins are too high, the translocon can become saturated and the recombinant protein may be retained in the cytoplasm. To determine if expression plasmids with a TIR^EVOLVED^ would saturate the Sec translocon we monitored production over a 5-h period, as well as with a higher (0.5 mM) IPTG concentration (Fig. 3a). We observed higher production levels of β-lactamase at a higher IPTG concentration (data not shown). And at all but one late time-point, a TIR^EVOLVED^ secreted more periplasmic β-lactamase than the corresponding TIR^UNEVOLVED^ (Fig. 3b). This observation was made at both low and high concentrations of IPTG. These time-course experiments therefore indicated that the Sec translocon was able to cope with the increased synthesis levels that were reached using a TIR^EVOLVED^.

### A TIR^EVOLVED^ can be used as a generic solution

**Fig. 4:**
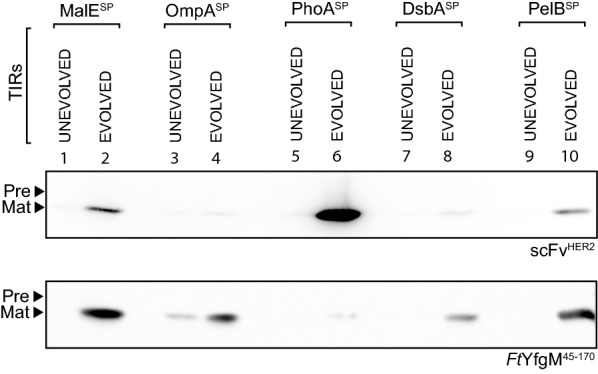
A TIR^EVOLVED^ is transferable. **a** Expression levels of scFv^HER2^ and *Ft*YfgM^45−170^ using five different signal peptides. In each instance TIR^UNEVOLVED^/TIR^EVOLVED^ pairs were assessed by immuno-blotting. The TIR^EVOLVED^ had originally been selected for β-lactamase (see Fig. 2). In this experiment, protein production was induced for 2 h, then a volume of cells corresponding to 0.2 OD_600_ units of cells were harvested, separated by a 12% SDS-PAGE and protein levels were determined by immunoblotting with antisera to a poly-histidine tag. ‘Pre’ denotes the precursor form of the protein and ‘Mat’ denotes the mature version

The five TIRs^EVOLVED^ presented in the previous section were easily selected because β-lactamase is compatible with high-throughput screening. High-throughput screening can be more challenging with other recombinant periplasmic proteins, so it might not be feasible to screen a large library for a TIR^EVOLVED^. We therefore asked whether a TIR^EVOLVED^ that we had selected using β-lactamase could be used to increase the production of other recombinant proteins in the periplasm of *E. coli*. In this set of experiments the coding sequences of scFv^HER2^ and *Ft*YfgM^45−170^ were expressed as fusions to the original five signal peptides, using both the TIR^UNEVOLVED^ and TIR^EVOLVED^ pairs. The expression plasmids were again transformed into BL21(*DE3*) *pLysS* and production was monitored using a mild induction protocol (0.05 mM IPTG for 2 h at 30 °C). As we had observed for β-lactamase, the TIR^EVOLVED^ always produced more protein than the corresponding TIR^UNEVOLVED^ (Fig. 4). Note that production from each TIR^UNEVOLVED^ was undetectable on these blots because the difference from the TIR^EVOLVED^ was too large to capture. We still noted that signal peptide performance was varied; the most effective signal peptide for production of scFv^HER2^ was PhoA^SP^, whilst the most effective for *Ft*YfgM^45−170^ was MalE^SP^. Thus, signal peptide performance might partly be explained by compatibility of the signal peptide.

**Fig. 5:**
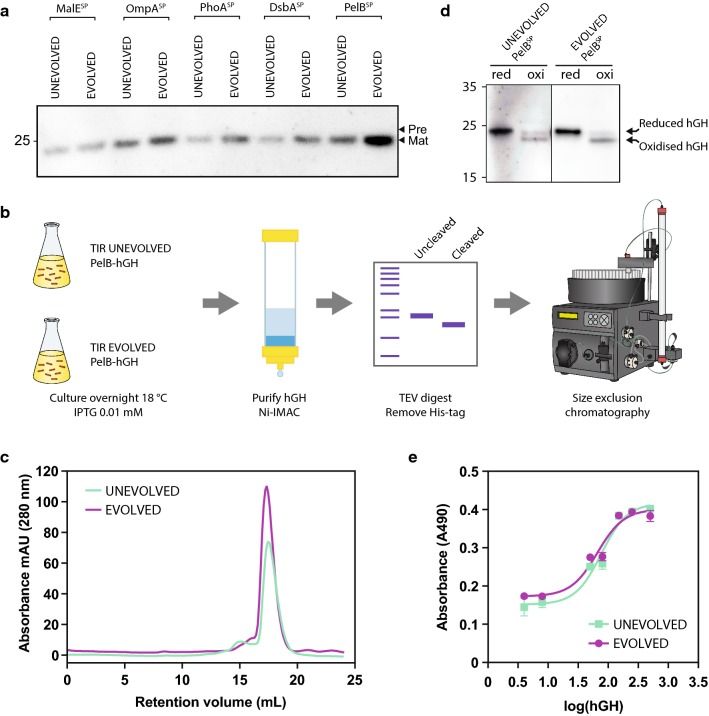
Production and purification of the human growth hormone (hGH) using a TIR^EVOLVED^. **a** Production levels of hGH using five different signal peptides. In each instance the difference between TIR^UNEVOLVED^/TIR^EVOLVED^ pairs was assessed by immuno-blotting. The TIR^EVOLVED^ had originally been selected for β-lactamase (see Fig. 2). In this experiment, protein production was induced for 2 h, then a volume of cells corresponding to 0.2 OD_600_ units of cells were harvested, separated by a 12% SDS-PAGE and protein levels were determined by immuno-blotting with antisera to a poly-histidine tag. ‘Pre’ denotes the precursor form of the protein and ‘Mat’ denotes the mature version. **b** An overview of the methodology used to purify hGH. **c** Analysis of the purified hGH by Size-Exclusion Chromatography (SEC). **d** Purified hGH was analysed by SDS-PAGE under denaturing- and non-denaturing conditions. **e** Activity of the purified hGH by using the MTS cell proliferation assay

A similar approach was taken to produce the human growth hormone (hGH). Here we observed that the most effective TIR^EVOLVED^ for production of hGH was the one coupled to the PelB^SP^ (Fig. 5a). To assess how much more protein was produced the N-terminally His-tagged hGH was purified by Immobilised Metal Affinity Chromatography (IMAC), the His-tag removed by proteolytic processing, and the sample polished by Size Exclusion Chromatography (SEC) (Fig. 5b). The yield of purified hGH was more than threefold higher using the TIR^EVOLVED^ compared to the TIR^UNEVOLVED^ (2.56 mg/L vs 0.79 mg/L). Importantly, we could not detect any difference in the quality of the purified hGH, as judged by monodispersity of the sample following SEC (Fig. 5c), the proportion of protein that had formed disulphide bonds (Fig. 5d), or the activity of the protein when tested by the MTS cell proliferation assay (Fig. 5e).

Taken together, this series of experiments indicate that the pET28a-based vectors containing signal peptides with an embedded TIR^EVOLVED^ can be used as a generic solution to increase production of single chain antibody fragments, hormones and other recombinant proteins in the periplasm of *E. coli* without compromising protein quality.

## Discussion

Why does a given signal peptide support a high-level of synthesis and secretion for one recombinant protein but not another? In this study we compared production levels of recombinant periplasmic proteins using different signal peptides. Initially the TIR was generated by ad hoc cloning (TIR^UNEVOLVED^), but we also utilised a directed evolution to select the evolved TIRs (TIR^EVOLVED^). Although the different TIRs did not influence the amino acid sequence of the recombinant protein being produced, they did affect the production levels; in all cases increased production was observed with a TIR^EVOLVED^. The data demonstrate that the nucleotide sequence of the TIR contributes to signal peptide performance in bacterial cell factories. This conclusion is best exemplified by our experiments with the PelB^SP^, which gave the lowest levels of β-lactamase production from a TIR^UNEVOLVED^ but the highest when expressed from a TIR^EVOLVED^. PelB^SP^ had therefore been converted from a ‘poor-performing’ signal peptide to a ‘top-performing’ signal peptide without changing a single amino acid.

Two previous studies have noted that nucleotide changes in the signal sequence can influence signal peptide performance. Ng and Sarkar noted that synonymous changes to the Usp45sp signal peptide in *Lactococcus lactis* increased production levels of a nuclease and an amylase by approximately 15% [33]. And Punginelli and co-workers noted that non-synonymous nucleotide changes in the signal peptide of the Tat-dependent formate dehydrogenase increased production levels by up to 60-fold in *E. coli* [34]. Both of these studies suggested that increased production yields correlated with reduced mRNA structures in the TIR. We were unable to correlate production yields to predicted mRNA structures in our study (see also [29, 35]). Further work is required to understand how structures within the mRNA and interactions with the ribosome allow certain TIRs to support higher levels of recombinant protein production.

Is there more to understand about signal peptide performance? Although the performance of MalE^SP^, OmpA^SP^, PhoA^SP^, DsbA^SP^ and PelB^SP^ was improved by generating a TIR^EVOLVED^, we still noted differences in the production levels of the recombinant proteins. These differences could potentially be explained by (1) differences in the efficiency of translational elongation of the signal peptides, or (2) compatibility between the signal peptide and the recombinant protein. The latter point is raised because the signal peptide acts to slow folding of the protein in the cytoplasm [16] so it could be expected that the physico-chemical characteristics of the signal peptide could impact on their performance. We therefore believe there is more to understand about ‘fitting’ of signal peptides with recombinant proteins. Until that point in time, there is still merit in testing a small selection of different signal peptides.

Improving signal peptide performance by optimisation of the TIR is a simple and inexpensive way to increase the production yields of secreted proteins in bacterial cell factories. It will be compatible with other published methods; such as those that use titratable promoters to tune transcription rates of secreted proteins [36]. At present there are no general rules for identifying an optimal TIR. It is therefore common practice to use bioinformatic prediction programs, such as RBS calculator [37], UTR designer [38] and RBS designer [39]. Herein we used a directed evolution approach to screen large TIR^LIBRARIES^. We were able to screen these TIR^LIBRARIES^ as we fused signal peptides to the β-lactamase protein, which confers resistance to β-lactam antibiotics. For proteins where no simple screening assay is available it is possible to directly couple [18], or translationally-couple β-lactamase to the recombinant protein [40–42]. It is also possible to use the pET28a-based TIRs^EVOLVED^, which we identified in this study and which improved production yields of a single chain antibody fragment, a hormone and another recombinant protein in *Escherichia coli*. However there may be limitations if these TIRs are transferred to other expression vectors, where the 5′UTR differs. Thus if other expression vectors are being used, we believe it would be better to create and screen a bespoke TIR^LIBRARY^ as demonstrated here.

## Methods

### Molecular cloning

The sequences encoding MalE^SP^, OmpA^SP^, PhoA^SP^, DsbA^SP^, PelB^SP^, β-lactamase, hGH and *Ft*YfgM^45−170^ were chemically synthesised (Genscript, USA). The sequence encoding scFv^HER2^ was obtained from the pHP2-15 plasmid [43]. All sequences are listed in Additional file 1: Table 1. To generate expression clones, the coding sequences and the *pET28a* vector were amplified by PCR using the Q5 polymerase (New England Biolabs, UK). The coding sequences were then cloned between the *Nco*I and *Nde*I restriction enzyme sites using the Gibson cloning method. Enzymes used for Gibson cloning were obtained from New England Biolabs, UK.

### Synthetic evolution of the TIR

TIR^LIBRARIES^ were generated by amplifying the expression plasmids by PCR, using overlapping primers as previously described [29, 30]. The forward primer was approximately 45 nucleotides in length and was partly degenerate. The design enabled complete randomization of the six nucleotides upstream of the AUG start codon, and partial randomization of the six nucleotides downstream stream of the AUG start codon (synonymous codons only). The reverse primer was always the same sequence (5′-CTCCTTCTTAAAGTTAAACAAAATTATTTCTAGAGGGGAATTGTTATC-3′). It overlapped with the forward primer by 13 nucleotides thus allowing circularization of the PCR product by homologous recombination in *E. coli* MC1061. The PCR was carried out using the Q5 polymerase (New England Biolabs, UK) in a program that consisted of 94 °C for 5 min and then 30 cycles of 95 °C for 45 s, 48–68 °C for 45 s (using a gradient thermocycler), 72 °C for 6 min and a final elongation step of 72 °C for 5 min. Specific PCR products that were amplified at the lowest annealing temperature were treated with *Dpn*I, then transformed into chemically competent *E. coli* MC1061. The transformation was seeded into 100 mL of Luria–Bertani containing 50 μg/mL kanamycin and incubated overnight at 37 °C. Isolation of the TIR^LIBRARIES^ was carried out using ten E.N.Z.A DNA mini kit purification columns (Omega Biotek, USA) and pooling of the eluates.

TIR^LIBRARIES^ were screened by transforming chemically competent BL21(*DE3*) *pLysS* and identifying clones that survived on the highest concentration of ampicillin. Here 0.5 μg of the TIR^LIBRARY^ was transformed into 50 μL of chemically competent BL21(*DE3*) *pLysS* using standard protocols. The entire transformation was then seeded into 3 mL of LB containing 50 μg/mL kanamycin and 34 μg/mL chloramphenicol. Cultures were grown at 37 °C with shaking for 16 h. Cultures were then back-diluted (1:50) into 5 mL of LB containing 50 μg/mL kanamycin and 34 μg/mL chlormphenicol and incubated as before until an OD_600_ of ~ 0.3 was reached. Expression of the coding sequence was induced by streaking a volume of cells corresponding to 0.002 OD_600_ units on LB agar containing 0.05 mM isopropyl-β-d thiogalactopyranoside (IPTG) and increasing concentrations of ampicillin (100–5000 μg/mL). Note that kanamycin and chloramphenicol were omitted from the plates. The plates were then incubated for 16 h at 37 °C. Colonies formed at higher ampicillin concentrations were selected for further analysis and sequencing (Eurofins MWG operon, Germany).

### Immuno-blotting

Cultures were grown at 37 °C with shaking for 16 h, then back-diluted (1:50) into 5 mL of LB containing 50 μg/mL kanamycin and 34 μg/mL chloramphenicol and incubated as before until an OD_600_ of ~ 0.3 to 0.5 was reached. Expression of the coding sequence was induced with 0.05 mM IPTG for 2 h at 30 °C. A volume of cells corresponding to an OD_600_ of either 0.02 or 0.2 was harvested by centrifugation then resuspended in 2 × Laemlli loading buffer [125 mM Tris–HCl pH 6.8, 4% SDS, 3% Glycerol, 0.02% bromphenol blue, 20% β–mercaptoethanol]. Proteins were separated by 12% SDS-PAGE then transferred to a nitrocellulose membrane using a semi-dry transfer apparatus (Bio-Rad, USA). The nitrocellulose membranes were probed with an antibody against either β-lactamase (Thermo Scientific, USA) or the poly-histidine tag (His-Probe, ThermoFisher Scientific, USA). Binding was detected using anti-mouse IgG linked to horseradish peroxidase (GE healthcare, USA) and a SuperSignal West femto luminol/enhancer solution (ThermoFisher Scientific, USA). Luminescence emitting from the nitrocellulose membrane was detected using an Azure Biosystems c600 device.

### Disc diffusion assays

Cells were grown in LB containing 50 μg/mL kanamycin and 34 μg/mL chlormphenicol until an OD_600_ of ~ 0.3. A volume of cells corresponding to an OD_600_ of 0.002 was then plated onto LB agar (lacking all antibiotics). A sterile filter disc containing 2 mg ampicillin was then placed on top of the cells and the plates were incubated at 37 °C for 16 h. Zones of growth inhibition were measured using a standard ruler.

### Purification of hGH

Expression plasmids harboring pET28a *pelB*-*hGH* were transformed into the expression host BL21(DE3) pLysS and grown on LB agar plates containing 50 µg/mL kanamycin and 34 µg/mL chloramphenicol. Single colonies were used to inoculate 100 mL of LB plus antibiotics medium which was grown overnight at 37 °C with shaking at 180 RPM. Overnight pre-cultures were used to inoculate 2 L flasks containing 1 L of LB media plus antibiotics, to a starting OD_600_ of 0.05. Cultures were grown to an OD_600_ of 0.7, at which point, flasks were incubated on ice for 10 min. Induction proceeded with the addition of 0.01 mM IPTG and incubation for 16 h at 18 °C with shaking at 180 RPM. Cells were harvested for 20 min at 4000*g*. Cell pellets were resuspended in 50 mL suspension buffer [50 mM Tris pH 8.0, 500 mM NaCl, 20 mM imidazole pH 8.0 and 1x protease inhibitor cocktail (cOmplete, Roche, USA)]. Cell suspensions were homogenized with a glass dounce homogenizer followed by cell disruption using an Avestin emulsiflex C3 high-pressure homogenizer (Avestin, Canada). Cell debris was removed by centrifugation at 20,000*g* for 30 min. Samples were applied to 2.5 mL Ni-Sepharose (GE Healthcare) and batch incubated at 4 ℃ for 1 h on a benchtop roller. The column was washed with 20 column volumes (50 mL) of wash buffer (50 mM Tris pH 8.0, 500 mM NaCl and 50 mM imidazole pH 8.0), followed by elution with 30 mL of elution buffer (50 mM Tris pH 8.0, 500 mM NaCl and 500 mM imidazole pH 8.0). The elution fraction was concentrated and buffer exchanged (50 mM Tris pH 8.0, 150 mM NaCl and 20 mM imidazole) using a centrifugal filter with a nominal MWCO of 10 kDa (Amicon, Merck Millipore). The N-terminal his-tag was proteolytically removed with TEV protease (purified in-house) at a 1:10 weight ratio and allowed to incubate overnight at 4 °C. Samples were reverse Ni purified, concentrated and applied to size exclusion chromatography using a Superdex 200 10/300 GL column (GE Healthcare, Sweden) in 50 mM Tris pH 8.0 and 100 mM NaCl. Relevant fractions were pooled, and concentrated. Sample concentration was measured by the BCA protein assay kit (Pierce, ThermoFisher Scientific, USA) and protein quality assessed by SDS-PAGE. Calculation of final yield per liter was determined by accounting of final volume, final OD at the conclusion of expression, and final concentration of purified hGH.

### MTS cell proliferation assay

The breast cancer MCF7 cell line (ATCC) was maintained in RPMI-1640 medium containing 10% FBS, 2 mM glutamine and 1% penicillin streptomycin (Gibco/Thermo Fisher Scientific) at 37 °C in a humidified atmosphere at 5% CO_2_. Cell proliferation following titration of purified hGH was determined according to the CellTiter 96 AQueous Non-Radioactive Cell Proliferation assay (MTS) protocol (Promega). Briefly, 1 × 10^4^ MCF7 cells were seeded in triplicate, in 100 µL aliquots into 96 well plates, followed by serum starvation for 24 h, prior to commencing the proliferation assay. Serially diluted hGH was added to the medium at a final concentration ranging from 0 to 400 ng/mL. Cell proliferation was assessed after 48 h of incubation, by addition of MTS and the electron coupling reagent PMS. The conversion of MTS to formazan was measured by absorbance at 490 nm using a SpectraMax plate reader. Background absorbance was corrected by subtraction of wells containing RPMI. hGH EC50 was calculated using GraphPad Prism 8.1.0.

## Supplementary information

**Additional file 1: Fig.** **1.** Selection of clones from TIR^LIBRARIES^**. Figure** **2.** Expression levels of β-lactamase using TIRs selected from TIR^LIBRARIES^. **Table** **1.** Coding sequences used in this study

## Data Availability

All data generated or analysed during this study are included in this published article [and its additional files].
