# Supplementary material for: Increased production of periplasmic proteins in Escherichia coli by directed evolution of the translation initiation region
Source: Microb Cell Fact. 2020 Apr 7;19:85. doi: 10.1186/s12934-020-01339-8 (PMC7137448; doi:10.1186/s12934-020-01339-8)
Supplement: Supplementary file 1 — Additional file 1: Fig. 1. Selection of clones from TIRLIBRARIES. Figure 2. Expression levels of β-lactamase using TIRs selected from TIRLIBRARIES. Table 1. Coding sequences used in this study [file 12934_2020_1339_MOESM1_ESM.docx]

*Supplementary Information*

**Increased production of periplasmic proteins in *Escherichia coli***

**by directed evolution of the translation initiation**

Kiavash Mirzadeh ^1,2,#^, Patrick J. Shilling ^1,#^, Rageia Elfageih ^1^, Alister J. Cumming ^1^, Huanhuan L. Cui ^3^,

Maja Rennig ^4^, Morten H. H. Nørholm ^2,4^, and Daniel O. Daley ^1,2^

^1^ Department of Biochemistry and Biophysics, Stockholm University, Sweden; ^2^ CloneOpt AB, Sweden; ^3^ Department of Medicine (Solna), Division of Microbial Pathogenesis, BioClinicum, Karolinska Institutet, Stockholm, Sweden; ^4^ Novo Nordisk Foundation Center for Biosustainability, Technical University of Denmark, Denmark.

# KM and PJS contributed equally to the work

Address correspondence to DOD, tel: +46 8 162 910, e-mail: [ddaley@dbb.su.se](mailto:ddaley@dbb.su.se)

**
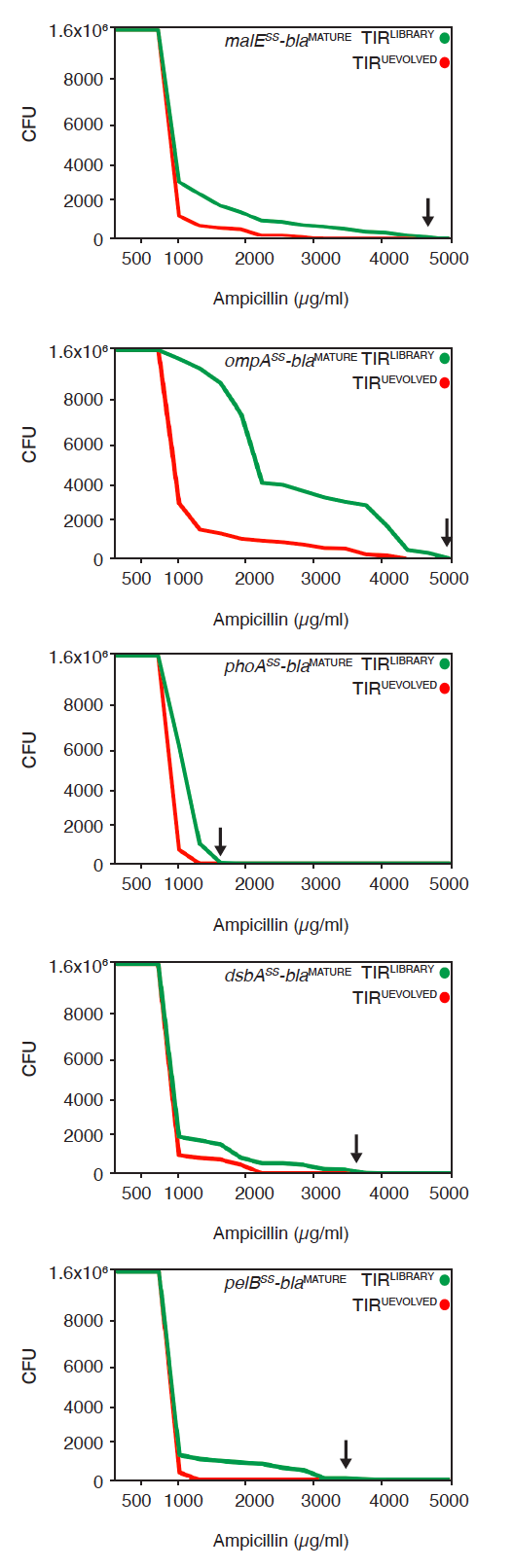
**

**Supplementary Figure 1. Selection of clones from TIR^LIBRARIES^.** TIR^LIBRARIES^ were transformed into BL21(*DE3*) *pLysS* and a volume corresponding to 0.002 OD_600_ units was plated on LB agar containing 0.05 mM isopropyl-β-D thiogalactopyranoside (IPTG) and increasing concentrations of ampicillin (100–5000 μg/mL). Colony Forming Units (CFU’s) were manually counted and plotted. Twenty colonies formed at higher ampicillin concentrations (denoted by the arrow) were selected for sequencing. For comparison, CFU’s obtained from a TIR^UNEVOLVED^ are shown.

**
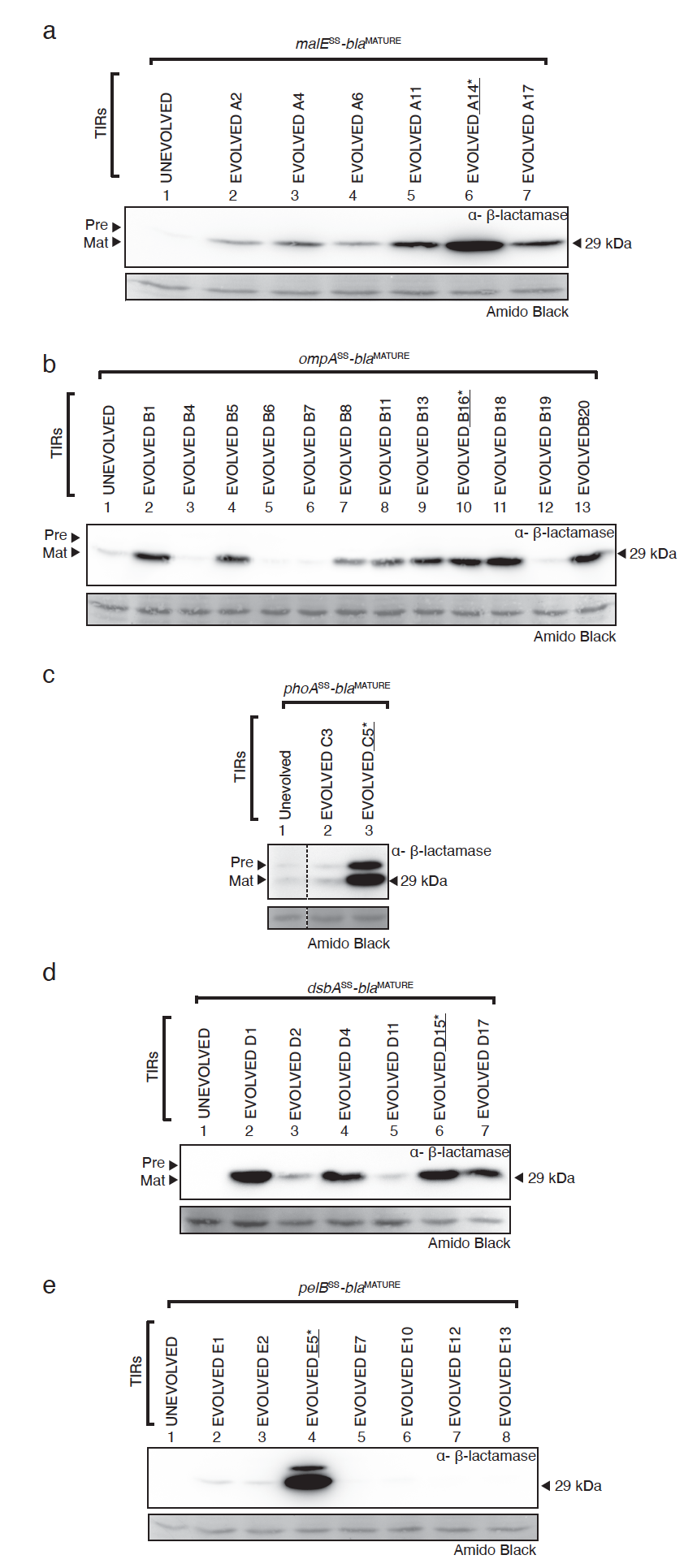
**

**Supplementary Figure 2. Expression levels of β-lactamase using TIRs selected from TIR^LIBRARIES^.** Twenty plasmids from the selection process (Supplementary Figure 1) were sequenced. Plasmids with a unique TIR (TIR^EVOLVED^) were compared to the TIR^UNEVOLVED^ by immuno-blotting. In this experiment, protein production was induced for two hours, then a volume of cells corresponding to 0.2 OD_600_ units of cells were harvested, separated by a 12 % SDS-PAGE and protein levels were determined by immunoblotting. A single TIR^EVOLVED^ was selected for further characterisation (denoted with an *).

**Supplementary Table 1. Coding sequences used in this study.**

MalE^SP^

ATGAAAATAAAAACAGGTGCACGCATCCTCGCATTATCCGCATTAACGACGATGATGTTTTCCGCCTCGGCTCTCGCC

OmpA^SP^

ATGAAAAAGACAGCTATCGCGATTGCAGTGGCACTGGCTGGTTTCGCTACCGTAGCGCAGGCC

PhoA^SP^

ATGAAACAAAGCACTATTGCACTGGCACTCTTACCGTTACTGTTTACCCCTGTGACAAAAGCC

DsbA^SP^

ATGAAAAAGATTTGGCTGGCGCTGGCTGGTTTAGTTTTAGCGTTTAGCGCATCGGCG

PelB^SP^

ATGAAATACCTGCTGCCGACCGCTGCTGCTGGTCTGCTGCTCCTCGCTGCCCAGCCGGCGATGGCC

β-lactamase

CACCCAGAAACGCTGGTGAAAGTAAAAGATGCTGAAGATCAGTTGGGTGCACGAGTGGGTTACATCGAACTGGATCTCAACAGCGGTAAGATCCTTGAGAGTTTTCGCCCCGAAGAACGTTTTCCAATGATGAGCACTTTTAAAGTTCTGCTATGTGGCGCGGTATTATCCCGTGTTGACGCCGGGCAAGAGCAACTCGGTCGCCGCATACACTATTCTCAGAATGACTTGGTTGAGTACTCACCAGTCACAGAAAAGCATCTTACGGATGGCATGACAGTAAGAGAATTATGCAGTGCTGCCATAACCATGAGTGATAACACTGCGGCCAACTTACTTCTGACAACGATCGGAGGACCGAAGGAGCTAACCGCTTTTTTGCACAACATGGGGGATCATGTAACTCGCCTTGATCGTTGGGAACCGGAGCTGAATGAAGCCTTACCAAACGACGAGCGTGACACCACGATGCCTGCAGCAATGGCAACAACGTTGCGCAAACTATTAACTGGCGAACTACTTACTCTAGCTTCCCGGCAACAATTAATAGACTGGATGGAGGCGGATAAAGTTGCAGGACCACTTCTGCGCTCGGCCCTTCCGGCTGGCTGGTTTATTGCTGATAAATCTGGAGCCGGTGAGCGTGGGTCTCGCGGTATCATTGCAGCACTGGGGCCAGATGGTAAGCCCTCCCGTATCGTAGTTATCTACACGACGGGGAGTCAGGCAACTATGGATGAACGAAATAGACAGATCGCTGAGATAGGTGCCTCACTGATTAAGCATTGGTAA

scFv^HER2^

GAAGTGCAGCTGGTCGAATCGGGTGGCGGATTAGTGCAGCCTGGAGGCTCCTTACGCCTGAGCTGTGCAGCGAGCGGCTTCAACATCAAGGACACCTACATACATTGGGTTCGCCAAGCTCCGGGCAAAGGTCTGGAGTGGGTTGCTCGTATCTATCCCACTAATGGGTATACACGCTATGCCGATAGCGTGAAAGGCCGGTTTACCATTAGCGCCGATACGAGCAAGAATACGGCGTATCTGCAGATGAACTCTCTGCGTGCCGAAGATACAGCGGTCTACTACTGCTCTCGTTGGGGTGGTGACGGGTTTTATGCAATGGACTATTGGGGCCAAGGAACCCTCGTGACGGTTTCCTCAGGCGGAGGTGGTAGTGGTGGCGGTGGGTCTGGCGGCGGTGGGAGCGACATTCAGATGACGCAGTCACCATCGTCGTTGTCAGCGTCGGTAGGTGATCGCGTCACGATTACCTGTCGTGCATCCCAAGATGTGAACACTGCAGTAGCGTGGTACCAGCAGAAACCGGGGAAAGCTCCGAAACTTCTGATTTACTCGGCGAGTTTCCTGTATAGTGGCGTTCCAAGTCGCTTTAGCGGTTCCCGTTCTGGCACGGATTTCACACTGACCATCTCAAGCTTGCAGCCGGAAGATTTTGCCACCTATTACTGCCAACAGCACTATACCACTCCTCCGACCTTTGGCCAAGGCACCAAAGTGGAGATCAAACGCCTAGGTGATTATAAAGACCATGACGGTGATTATAAAGATCATGACATCGATTACAAGGATGACGATGACAAGGCGGCCGCCCATCATCATCATCATCAT

Blue = 3x flag tag

Green = His tag

*Ft*YfgM^45-170^

CATCACCATCACCATCACCACCACGAGAATTTGTATTTTCAGGGTCATATGACCATCTATCAGAAGGCCTTGATCGCCAACGAAAACCCAAAAAGCAGTGTGGAGACCAAGATCGCGAAATTCGAACAGGTGGTGAACGACTATCCTAATACCTCGTTCGGCATTTTCGCTTCATGGCAGCTCGCGGATTTGTATACCACGCCGACCAAGCCAGACAGCAAGAATTTCAACGTGAATATCACCAATTTACCGAAAGCCATCGCCATCCTTCAGCAGTCCATTGAAAACAACCCTAAAGATTCGCTGTCCGATATTTCCAAGGTCCGTCTGGCTCGTCTCTATATCGTCGCGAAGCAGCCAGATCAGGCGATTAAAACTCTGCAGGGCATCAAATCGTTTAAGGATAACGCATATCCTCTGATGCTGTTAGGTCAGGCTTACTCGGAAAAAAAAGATAAAGTAAAGGCAATTGAATCGTGGCAGAAGGCCCTGCAAGACCCTAACTCTTCTGACCAATTCAAGCAGATTATCAGCCAGCTGATTAACAACACAAATTAATAA

Green = His tag

Yellow = TEV protease site

hGH

CATCACCATCACCATCACCACCACGAGAATTTGTATTTTCAGGGTTTTCCGACCATCCCGCTGAGCCGTCTGTTTGACAATGCGATGCTGCGTGCGCACCGTCTGCACCAACTGGCGTTTGACACCTACCAAGAGTTCGAGGAAGCGTACATCCCGAAGGAACAGAAATATAGCTTCCTGCAGAACCCGCAAACCAGCCTGTGCTTTAGCGAGAGCATTCCGACCCCGAGCAACCGTGAGGAAACCCAGCAAAAGAGCAACCTGGAGCTGCTGCGTATCAGCCTGCTGCTGATTCAGAGCTGGCTGGAACCGGTGCAATTCCTGCGTAGCGTTTTTGCGAACAGCCTGGTGTACGGCGCGAGCGACAGCAACGTTTATGACCTGCTGAAGGATCTGGAGGAAGGTATCCAAACCCTGATGGGTCGTCTGGAAGACGGCAGCCCGCGTACCGGTCAGATTTTCAAGCAAACCTACAGCAAATTTGATACCAACAGCCACAACGACGATGCGCTGCTGAAAAACTACGGCCTGCTGTATTGCTTTCGTAAGGACATGGATAAAGTTGAGACCTTCCTGCGTATCGTTCAGTGCCGTAGCGTTGAGGGTAGCTGCGGTTTCTAA

Green = His tag

Yellow = TEV protease site
